# Quantitative Proteomics Reveals the Defense Response of Wheat against *Puccinia striiformis* f. sp. *tritici*

**DOI:** 10.1038/srep34261

**Published:** 2016-09-28

**Authors:** Yuheng Yang, Yang Yu, Chaowei Bi, Zhensheng Kang

**Affiliations:** 1 College of Plant Protection, Southwest University, Beibei, Chongqing, 400715, P. R. China; 2 Key Laboratory of Plant Protection Resources and Pest Management of Ministry of Education, State Key Laboratory of Crop Stress Biology for Arid Areas and College of Plant Protection, Northwest A&F University, Yangling, Shaanxi, 712100, P. R. China

## Abstract

Wheat stripe rust, caused by *Puccinia striiformis* f. sp. *tritici (Pst*), is considered one of the most aggressive diseases to wheat production. In this study, we used an iTRAQ-based approach for the quantitative proteomic comparison of the incompatible *Pst* race CYR23 in infected and non-infected leaves of the wheat cultivar Suwon11. A total of 3,475 unique proteins were identified from three key stages of interaction (12, 24, and 48 h post-inoculation) and control groups. Quantitative analysis showed that 530 proteins were differentially accumulated by *Pst* infection (fold changes >1.5, *p* < 0.05). Among these proteins, 10.54% was classified as involved in the immune system process and stimulus response. Intriguingly, bioinformatics analysis revealed that a set of reactive oxygen species metabolism-related proteins, peptidyl–prolyl *cis–trans* isomerases (PPIases), RNA-binding proteins (RBPs), and chaperonins was involved in the response to *Pst* infection. Our results were the first to show that PPIases, RBPs, and chaperonins participated in the regulation of the immune response in wheat and even in plants. This study aimed to provide novel routes to reveal wheat gene functionality and better understand the early events in wheat–*Pst* incompatible interactions.

Rust fungi are a monophyletic group of obligate biotrophic parasites that invade and cause diseases in economically important plants^1^,^2^. Species of rusts have evolved such that the pathogens are highly specific to the plant species they can infect, colonize, and reproduce^3^. A well-known representative of rust fungus is *Puccinia striiformis* f. sp. *tritici (Pst*), the causal agent of the wheat stripe rust disease, which is considered one of the most aggressive diseases in wheat production^4^. As an economically important pathogen, remarkable progress in stripe rust resistance genes and molecular perspectives has been made in the interaction between *Pst* and wheat^4^. However, the frequent virulence variation of *Pst* races often overcome the race-specific resistance of hosts and negate the breeders’ efforts, which has been a prominent question for durable disease control. Moreover, gene expression is regulated at different levels by multifactorial and system-level approaches; consequently, the DNA sequence is insufficient to elucidate sophisticated immune interaction characteristics^5^. Therefore, the development of novel approaches is necessary to further understand the resistance mechanisms of wheat response against *Pst*.

Proteomics has emerged as complementary to genomics and transcriptomics because it focuses on gene products, thereby providing a more direct view of cellular immunological processes than genomics or transcriptomics^6^,^7^. Proteomics offers the possibility of simultaneously studying protein localization, protein–protein interactions, enzymatic complexes, or post-translational modifications that are essential to better understand plant–pathogen interactions^8^. To date, proteomics based on mass spectrometry (MS) has matured and become a powerful “hypothesis-generating engine” that provides a framework for translating large data sets to understand complex biological processes^9^,^10^,^11^.

Extensive quantitative proteomic studies with high-throughput proteome research techniques have been conducted on plant–pathogen interactions, such as Arabidopsis*–Pseudomonas syringae*^12^, tomato–*P. syringae*^13^, potato–*Phytophthora infestans*^14^, and *Zantedeschia aethiopica*–*Pectobacterium carotovorum*^15^. However, quantitative proteomic research has rarely examined the changes in the wheat proteome in response to biotrophic fungi (especially to rust fungi); only a few studies based on gel electrophoresis methods are available^16^,^17^. The key issue for this shortcoming is that the allohexaploid wheat genome consists of three closely related sub-genomes (A, B and D)^18^, which greatly influenced the research on wheat functional genomics and proteomics.

To further clarify the resistance mechanism of wheat against *Pst* at the proteomic level in the current study, the quantitative proteome of the wheat cultivar Suwon11 (Su11) was compared between plants inoculated or uninoculated with the avirulent *Pst* race CYR23 *in planta*. Results showed that 530 proteins were differentially expressed by *Pst* infection. Notably, a set of reactive oxygen species (ROS) metabolism-related proteins, peptidyl–prolyl *cis–trans* isomerases (PPIases), RNA-binding proteins (RBPs), and chaperonins was revealed to be involved in the response to *Pst* infection for the first time. This study provides a novel route and a theoretical basis to further clarify the molecular mechanism and defense network of wheat response against *Pst*.

## Results

### Overview of the proteome identification of wheat–*Pst* incompatible interaction

**Figure 1:**
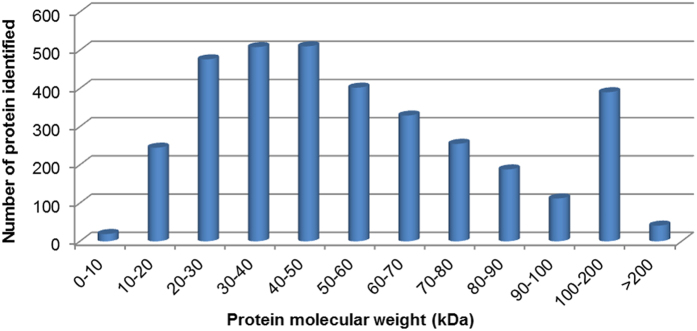
Molecular mass distribution of the wheat proteins identified from the iTRAQ analysis spanned across a wide range of molecular weights, which were induced by incompatible *Puccinia striiformis* f. sp. *tritici*. The abscissa represented the molecular weight of identified proteins (kDa), and the ordinate represented the number of identified proteins.

**Figure 2:**
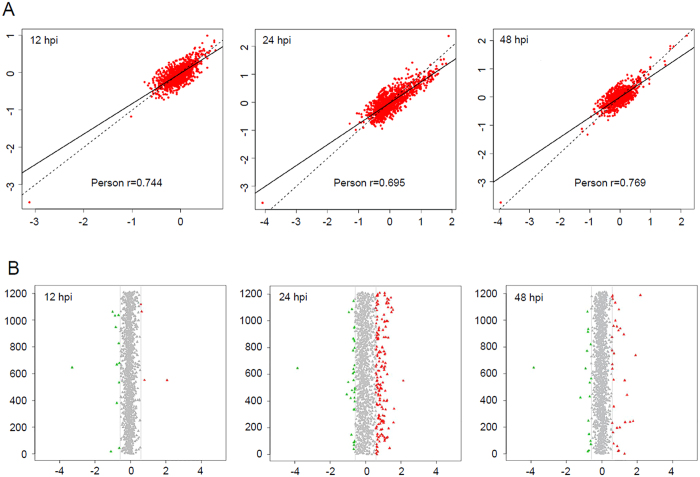
The quantitative proteomic analysis of the wheat peoteins induced by incompatible *Puccinia striiformis* f. sp. *tritici*. (**A**) The correlation of three treatment groups (12, 24 and 48 hpi). The Pearson correlation factors are 0.744, 0.695 and 0.769, respectively. The abscissa represented the first repeat whereas the ordinate represented the second repeat (as log_2_ value). hpi, hour post-inoculation. (**B**) Gaussian distribution of the quantitative dates of three treatment groups (12, 24, and 48 hpi). The ordinate represented the quantity of identified proteins, and the abscissa represented protein ratios (as log_2_ value). Red triangles indicated up-regulated proteins whereas green triangles indicated down-regulated proteins. hpi, hour post-inoculation.

Wheat leaf samples from 12, 24, and 48 h post-inoculation (hpi) treatments or water-inoculated controls were collected and analyzed by iTRAQ. After labeling, SCX fractionation, and LC-ESI-MS/MS analysis, a total of 3475 proteins were identified from the wheat–*Pst* incompatible interaction. Among these proteins, 1774, 338, and 1363 wheat proteins were obtained from three separate genome sequence databases of *Aegilops tauschii* (the wheat D genome progenitor), *Triticum urartu* (the wheat A genome progenitor), and the hexaploid *Triticum aestivum* cultivar Chinese Spring, respectively (Table 1 and S1). The mass distribution of each identified protein spanned across a wide range of molecular weights higher than 10 kDa (Fig. 1). A data correlation analysis of the 3475 proteins showed the Pearson correlation coefficient values of 0.744, 0.834 and 0.769, respectively among the replicates of three treatments compared to control (Fig. 2A). All the identified wheat proteins were analyzed by gene ontology (GO) and classified by the three ontologies (cellular component, biological process, and molecular function). After excluding the GO entries without a corresponding protein, all the identified proteins were associated with 49 GO categories (Figure S1).

### Bioinformatics analysis of the regulated proteins

**Figure 3:**
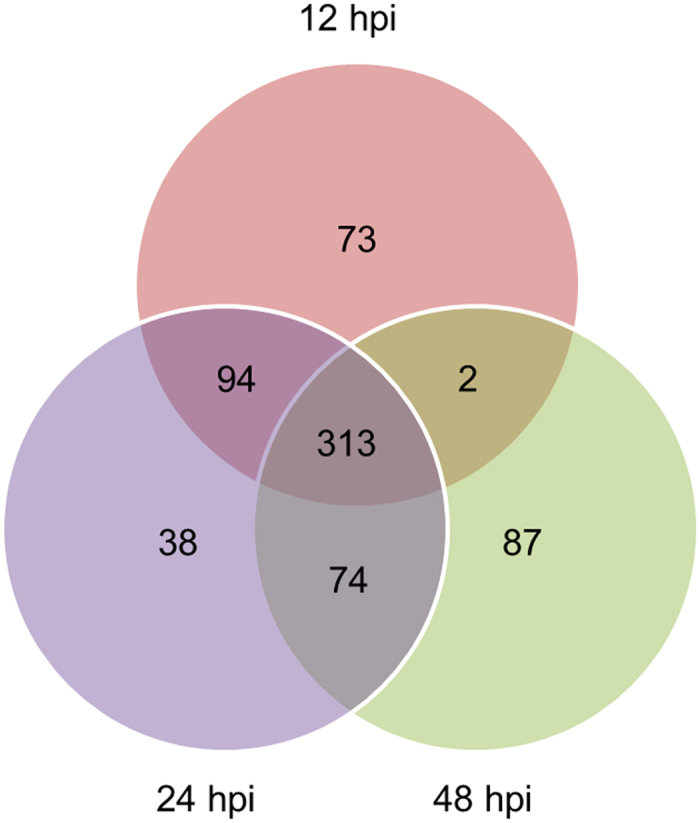
Venn diagram representing the overlap among differentially expressed proteins identified by iTRAQ analysis of three treatment groups (12, 24, and 48 hpi) of wheat–*Puccinia striiformis* f. sp. *tritici* incompatible interaction. hpi, hour post-inoculation.

To reveal the proteins with a putative regulatory function in the wheat–*Pst* incompatible interaction, the Gaussian distributions of the quantitative ratio (as log_2_ value) were performed (Fig. 2B). According to the means and the standard deviation values of the Gaussian distribution, there were a combined total of 530 wheat proteins with significantly altered expression in the 12, 24, and 48 hpi treatments compared with the controls by a fold-change > 1.5 (*P* < 0.05) (Fig. 3). Among these regulated proteins, 279 proteins were identified from *Ae. tauschii*, 56 proteins were identified from *T. urartu*, and 195 proteins were identified from *T. aestivum* (Table S2, Figure S2). Compared with control group, there were 171, 29 and 122 up-regulated proteins and 58, 17 and 35 down-regulated proteins in the 24 hpi treatment between *Ae. tauschii*, *T. urartu*, and *T. aestivum*, respectively (Table 2). The subsequent Kyoto Encyclopedia of Genes and Genomes pathway analyses categorized the differentially accumulated proteins to 84 respective pathways, and the proteins were mainly involved in the metabolic pathways (26.9%), biosynthesis of secondary metabolites (17.4%), and ribosome (9.5%).

**Figure 4:**
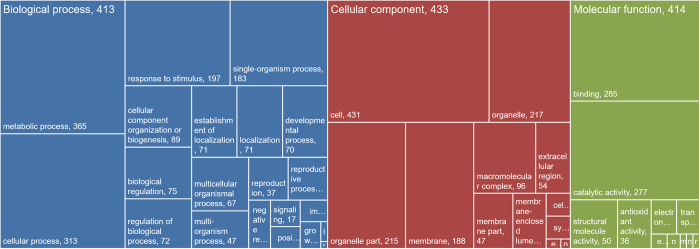
Gene Ontology annotation of differentially expressed proteins identified by iTRAQ analysis in the wheat–*Puccinia striiformis* f. sp. *tritici* incompatible interaction. GO enrichment analysis of identified proteins by Blast2GO software, the three unrelated ontologies: biological process, cellular component and molecular function were analyzed, respectively.

All 530 significantly accumulated proteins identified were analyzed for gene ontology using the Blast2GO software, and classified by the three unrelated ontologies (Fig. 4). For each of these three ontologies, annotated data revealed that these proteins are mainly distributed among two or three of the general term categories: within the 413 proteins (77.9%) involved in biological processes, 365 (88.4%) and 313 (75.8%) are dedicated to metabolic processes and cellular processes, respectively; within the 433 proteins (81.7%) classified in cellular components sub-ontology, 431 (99.5%) and 217 (50.1%) are related to cell and organelle components, respectively; similarly, in molecular functions sub-ontology, 285 (68.8%) and 277 (66.9%) of the 414 proteins (78.1%) have binding and catalytic activity, respectively (Fig. 4).

### Remarkable differentially accumulated protein groups in response to *Pst* infection

#### ROS metabolism-related proteins

**Figure 5:**
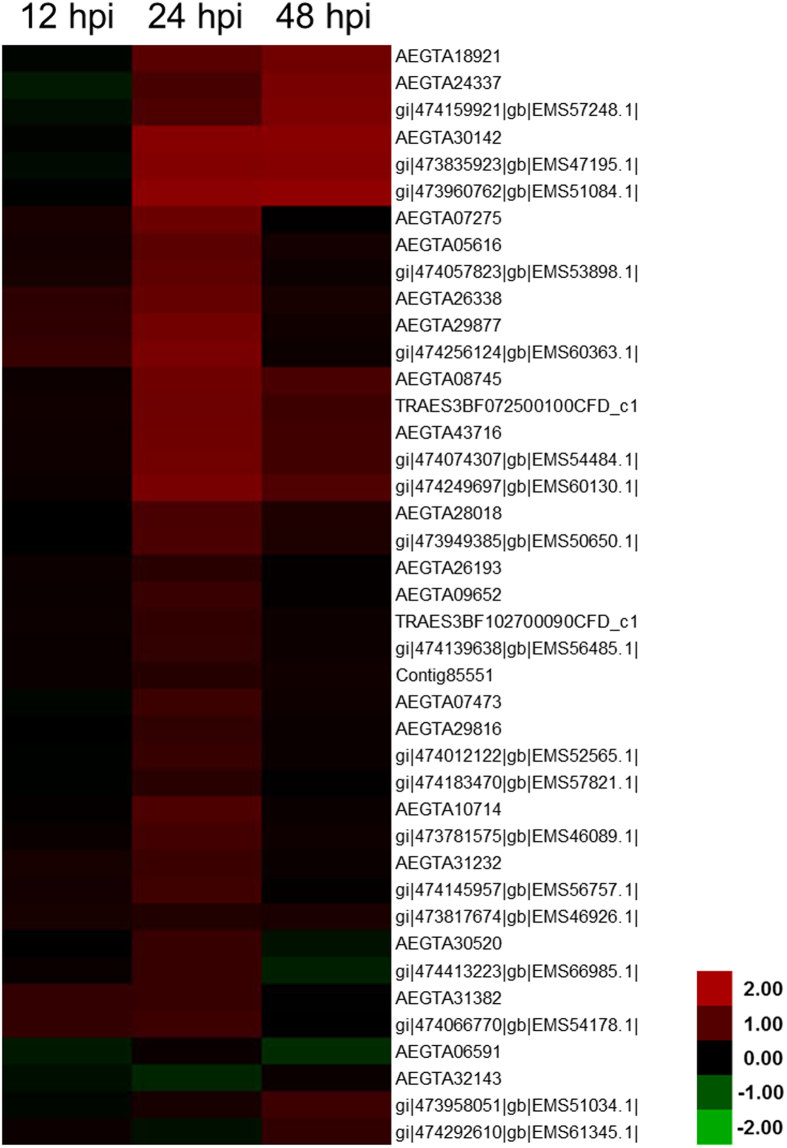
Heat map representing the profile of differentially accumulated reactive oxygen species metabolism-related proteins induced by the avirulent *Puccinia striiformis* f. sp. *tritici* race CYR23. Red color indicated high expression whereas green color indicated low expression.

Among the affected wheat proteins in response to *Pst* infection, 42 proteins associated with ROS metabolism were significantly accumulated (Table 3). These accumulated proteins included superoxide dismutase, ascorbate peroxidase (APX), catalase, glutathione peroxidase (GPX), and peroxiredoxin. Compared with the water-inoculated control, differential expression analysis indicated that all these proteins were up-regulated in different levels at the three time points, especially in 24 hpi (Fig. 5). In particular, 11 peroxidases were strongly induced in both 24 and 48 hpi. These results further demonstrated that ROS metabolism-related proteins, especially peroxidases, played a positive role in the defense response against *Pst* infection.

#### PPIases

**Figure 6:**
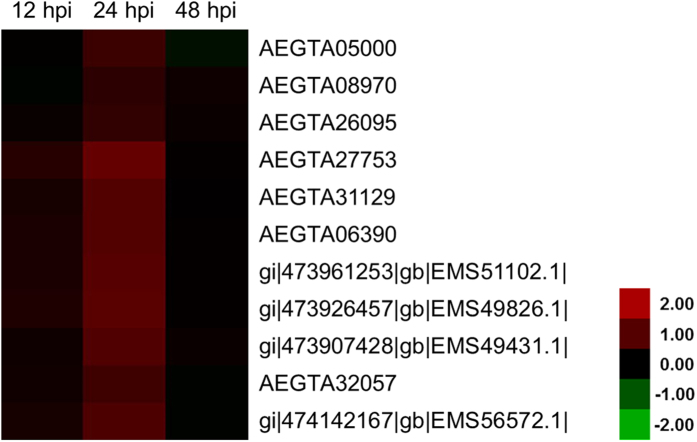
Heat map representing the profile of differentially accumulated wheat peptidyl–prolyl *cis–trans* isomerases induced by the avirulent *Puccinia striiformis* f. sp. *tritici* race CYR23. Red color indicated high expression whereas green color indicated low expression.

Bioinformatics analysis also indicated that 12 regulated proteins were involved in the defense against *Pst* (Fig. 6). COG functional description showed that these proteins were PPIases. COG analysis also categorized seven of these PPIases as the FKBP type (immunophilins that bind with FK-506), whereas the remaining five belonged to the cyclophilin family (immunophilins that bind with cyclosporine A). Consequently, these 12 PPIases were also strongly up-regulated at 24 hpi. All cyclophilin family proteins were simultaneously up-regulated in three different treatments with different levels (Fig. 6).

#### RBPs

**Figure 7:**
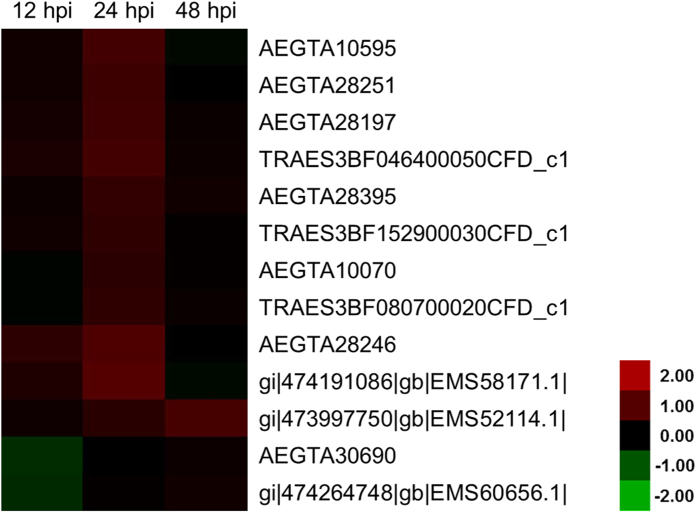
Heat map representing the profile of differentially accumulated wheat RNA-binding proteins induced by the avirulent *Puccinia striiformis* f. sp. *tritici* race CYR23. Red color indicated high expression whereas green color indicated low expression.

As shown in Fig. 7, 13 proteins were categorized as RBPs and significantly up-regulated during the incompatible interaction. Therefore, these RBPs were associated with the stress response of *Pst* infection. These RBPs included one alternative splicing regulator (AEGTA28251), one arginine/serine-rich splicing factor (TRAES3BF080700020CFD_c1), two predicted glycine-rich RBPs (AEGTA28395 and TRAES3BF152900030CFD_c1), two eukaryotic translation initiation factor (AEGTA30690 and gi|474264748|gb|EMS60656.1|), and the remaining seven RBPs were described as predicted proteins containing RNA recognition motifs. Except for AEGTA30690, all the selected proteins had a significantly altered level of response in 24 hpi (Fig. 7).

#### Chaperonins

**Figure 8:**
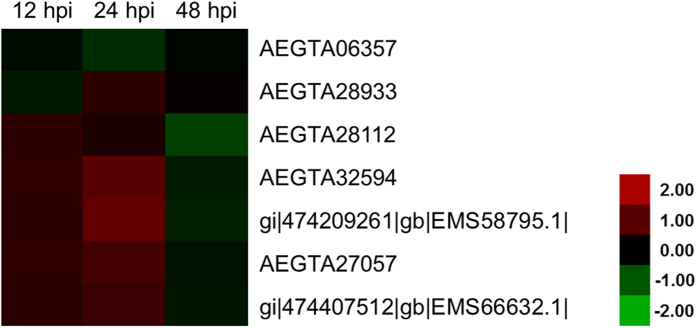
Heat map representing the profile of differentially accumulated wheat chaperonins induced by the avirulent *Puccinia striiformis* f. sp. *tritici* race CYR23. Red color indicated high expression whereas green color indicated low expression.

According to the COG functional description, seven proteins were identified and annotated as chaperonins based on their expression profiles after *Pst* inoculation (Fig. 8). Among the chaperonins, six (AEGTA27057, AEGTA28112, AEGTA28933, AEGTA32594, gi|474209261|gb|EMS58795.1|, and gi|474407512|gb|EMS66632.1|) were obviously up-regulated at 24 hpi, but one of the chaperonins (AEGTA06357) was down-regulated at the three different time points (Fig. 8). These results were consistent with the COG functional description, which categorized the six up-regulated proteins into the same type as the co-chaperonin GroES (HSP10), and the down-regulated one as the chaperonin GroEL (HSP60 family). These results suggested that the co-chaperonin GroES (HSP10) was likely to play crucial roles in the defense response of rust fungus.

### Validation of differentially accumulated proteins by qRT-PCR

**Figure 9:**
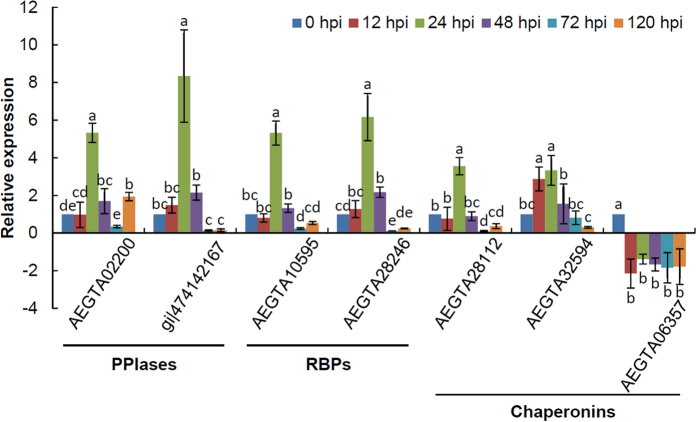
Expression patterns of differentially expressed proteins induced by the avirulent *Puccinia striiformis* f. sp. *tritici* race CYR23. hpi, hour post-inoculation. Leaf tissues were sampled for both inoculated and mock-inoculated plants at 0, 12, 24, 48, 72 and 120 hpi. The relative expression levels of these genes were calculated using the comparative threshold (2^−ΔΔC^_T_) method. The mean value and standard deviation of gene expression were calculated from three independent biological replications. ANOVA was conducted to determine the differences between each time point. Superscripts with the same letter indicate that values are not significantly different at *p* < 0.01.

To further confirm the expression patterns of coding genes after incompatible *Pst* infection, qRT-PCR analysis was performed. Due to that ROS plays a vital role in plant immunity as well-known, seven coding genes of identified PPIases, RBPs and Chaperonins mentioned above were selected. As revealed in Fig. 9, the mRNA levels of six proteins of them exhibited significant rises (*p* < 0.01, fold−change > 3) in at least one sampling time point compared with the control, especially at 24 hpi. Exceptionally, the relative expression level of AEGTA06357 (HSP60 family) was significantly down-regulated (*p* < 0.01, Fig. 9). These remarkable changes were consistent with the induced accumulation of corresponding proteins and further supported the differentially expressed proteins identified by iTRAQ. Additionally, the mRNA level of gi|474142167|gb|EMS56572.1| was clearly up-regulated at 24 hpi, as compared with the control, with more than an 8-fold increase, while the mRNA level of AEGTA32594 was induced at as early as 12 hpi. AEGTA06357 expression was also suppressed as early as 12 hpi and maintained at the same level thereafter (Fig. 9).

### Response to *Pst* infection after knocking down the transcription of differentially accumulated proteins

**Figure 10:**
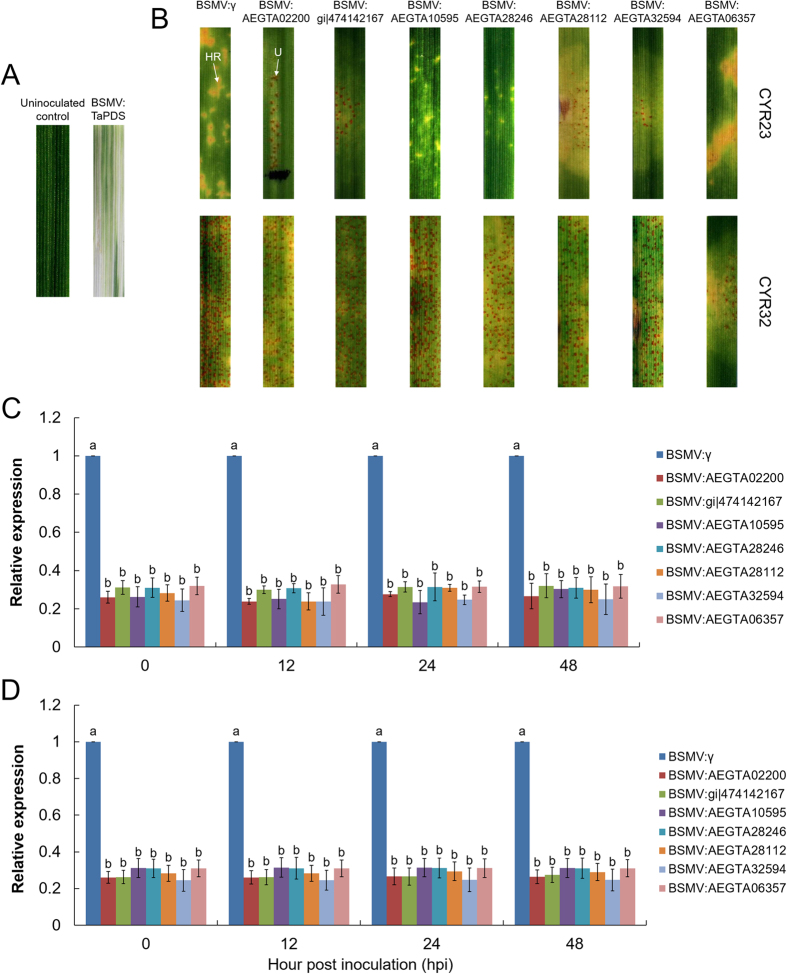
Functional characterization of differentially expressed proteins by BSMV-VIGS system. (**A**) Mild chlorotic mosaic symptoms were observed on the fourth leaves inoculated with BSMV:TaPDS at 12 dpi; uninoculated control, wheat leaves treated with full-strength inoculation buffer. (**B**) Disease symptoms were observed at 14 dpi on the fourth leaves of wheat plants that were inoculated with the avirulent pathogen CYR23 and virulent pathogen CYR32, respectively. HR, hypersensitive response; U, uredium. (**C**) Relative transcript levels of differentially expressed proteins assayed in knocked-down wheat leaves inoculation with CYR23. (**D**) Relative transcript levels of differentially expressed proteins assayed in knocked-down wheat leaves inoculation with CYR32. The mean value and standard deviation of gene expression were calculated from three independent biological replications. ANOVA was conducted to determine the differences between each time point. Superscripts with the same letter indicate that values are not significantly different at *p* < 0.01.

To further investigate the functions of seven selected proteins in response to *Pst* infection, the BSMV-VIGS system was employed to knock down the transcription of their coding genes. The feasibility and silencing efficiency of the BSMV-VIGS system was tested using the wheat phytoene desaturase (*TaPDS*) as a positive control. At 12 dpi with BSMV:TaPDS, obvious photo-bleaching was observed on wheat seedlings when *TaPDS* was silenced, indicating that the RNAi system is effective for assessing the potential roles of candidate genes (Fig. 10A).

Under the same conditions, all of the BSMV-inoculated plants displayed mild chlorotic mosaic symptoms at 12 dpi. Then the fourth leaves were inoculated with fresh urediniospores of *Pst* races CYR23 or CYR32, respectively. As shown in Fig. 9B, conspicuous HR was elicited by CYR23 on mock-inoculated plants or leaves that were previously infected with BSMV:γ, but various numbers of *Pst* uredia were produced on leaves infected with BSMV:AEGTA02200, BSMV:gi|474142167, BSMV:AEGTA28112 and BSMV:AEGTA32594. Although no uredium was observed on leaves infected with BSMV:AEGTA10595 and BSMV:AEGTA28246, whereas significantly less necrotic areas observed on their leaves compared with control plants at 14 dpi (Fig. 10B). In contrast, all leaves inoculated with CYR32 produced numerous uredia at 14 dpi, except that less *Pst* uredia were produced on leaves infected with BSMV:AEGTA06357 (Fig. 10B). Intriguingly, obviously necrosis was observed on the BSMV: AEGTA32594 plant leaf segment (Fig. 10B). Meanwhile, the transcription levels of all selected genes in the infected BSMV-inoculated leaves were lower than 33.0% after infection with *Pst* at 0, 12, 24 and 48 hpi, respectively (Fig. 10C,D). These observations are consistent with the qRT-PCR results and further confirmed that AEGTA02200, gi|474142167, AEGTA10595, AEGTA28246, AEGTA28112 and AEGTA32594 may be involved in the plant defense reaction, and AEGTA06357 may participated in the negative regulation of plant immunity against the stripe rust fungus.

## Discussion

Previous proteomic research on plant–microbe interactions was mostly implemented by gel electrophoresis-based methods (such as SDS-PAGE, 2-DE, and 2D-DIGE)^19^. However, the proteins obtained from these methods cannot be fully representative of the whole protein profiles at any specific period because of their limited sensitivity, resolution, and speed of data capture^20^. In the current study, we first performed *in planta* quantitative proteomic analysis of wheat in the immune response of *Pst* by an iTRAQ-based differential expression approach; iTRAQ is an easy and reliable technique for the quantitative investigation of proteomics based on chemical labeling with stable isotopes^21^. Compared with the three existing databases^22^,^23^,^24^, the proportion of proteins identified with the *Ae. tauschii* genome sequence was higher than those with *T. urartu* and *T. aestivum* (Table S1). Overall, we identified 530 differentially accumulated wheat proteins between the different treatments. According to Figure S1, 10.54% of the proteins for biological process was linked to the immune system process and stimulus response (such as chitinases, β-glucanases, and several defense response enzymes in Table 3). Nevertheless, we focused on several remarkable and pivotal protein groups and novel discoveries from the quantitative results.

### ROS-related proteins

In plants, ROS production is one of the earliest cellular responses following successful pathogen recognition. ROS act as executioners of pathogens, as well as signaling molecules involved in triggering the hypersensitive response (HR) and activating signal transduction processes to stop pathogen growth^25^,^26^,^27^. Previously, ROS generation was associated with hypersensitive cell death responses in the incompatible interaction between wheat and avirulent *Pst* races^28^. ROS are commonly generated by NADPH oxidases (also known as the respiratory burst oxidases) and peroxidases in plant cells^25^,^29^. NADPH oxidases were initially described in mammalian neutrophils and are located in the plasma membrane; these proteins correspond to one of the most studied systems that participate in ROS production to defend cells from invasion^25^,^30^. Notably, none of the NADPH oxidases were detected in our current results, but 16 peroxidases were identified and showed noticeably higher expression during infection than in the controls (including class III peroxidase, GPX, APX, and thioredoxin-dependent peroxidase; Table 3). These results suggested that peroxidases but not NADPH oxidases might play more important roles in the oxidative response of wheat to *Pst* invasion. Consistent with our results, Dmochowska-Boguta *et al.*^31^ proved that the induction of peroxidases is more pronounced than that of NADPH oxidases in wheat–*P. triticina* interactions, and postulated that class III peroxidases play a leading role in the formation of ROS molecules during the response of wheat to *P. triticina* infection. Moreover, a recent report showed that the wheat stripe rust resistance protein WKS1 is targeted to the thylakoid-associated ascorbate peroxidase to detoxify ROS^32^. All the aforementioned results revealed that the production of ROS in wheat against rust infection might be more dependent on peroxidases.

### PPIases

In prokaryotic and eukaryotic cells, PPIases form a superfamily of proteins for facilitating the *cis–trans* isomerization of N-terminal peptide bonds to proline residues within polypeptide chains^33^. These proteins were first identified in mammals as receptors of the immune-suppressing drug cyclosporine A. PPIases have been classified into three distinct families: the cyclosporin-binding cyclophilins (CyP), FK506-binding proteins (FKBP), and FK506- and cyclosporin-binding protein (FCBP)^34^. Several studies have demonstrated that PPIases can participate in plant immune processes. Godoy *et al.*^35^ found that *StCyP*, a *Solanum tuberosusm* cyclophilin gene, is involved in the response to *Fusarium solani* f. sp *eumartii* infection and environmental stresses. A pepper cyclophilin (*CACYP1*) mRNA is strongly induced by *Xanthomonas campestris* pv. *vesicatoria* and *Colletotrichum gloeosporioides* infections^36^. Park *et al.*^37^ purified and characterized a FKBP-type PPIase in Chinese cabbage, which showed *in vitro* antifungal activity against *Candida albicans*, *Botrytis cinerea*, *Rhizoctonia solani*, and *Trichoderma viride*. Wang *et al.*^38^ identified four differentially accumulated PPIases in rice stripe virus-infected rice leaves. Several *Arabidopsis* PPIase genes were also involved in the defense response to *P. syringae* and *X. campestris* invasion^39^,^40^. The present study provides the first evidence of the involvement of PPIases in the wheat response to obligate *Pst* infection. Among the eight induced wheat PPIases that we identified, five were annotated to the FKBP family, whereas the remaining three were annotated to the cyclophilin family (Table 3). Another recent study revealed that the *Arabidopsis* effector-triggered immune receptor RPM1 is activated via the bacterial effector AvrB-induced phosphorylation of the RPM1-interacting protein RIN4, whereas RIN4 is also regulated by PPIase ROC1-mediated isomerization^41^. Notably, one up-regulated protein (AEGTA03060) was identified and annotated as RPM1 in our results (Table 3). Therefore, further investigations are necessary to confirm whether wheat PPIases contribute to the immune response against *Pst* via a similar mechanism.

### RBPs

RBPs are proteins that bind to RNA molecules in cells and coordinate RNA processing and post-transcriptional gene regulation^42^. In mammals, sequence-specific RBPs play critical roles in the immune response to modulate the gene expression of target mRNAs^43^. Based on the latest evidence, RBPs are important regulators of plant immunity at each level of RNA processing^44^. In *Arabidopsis*, several RBPs have been implicated in the defense against viral and bacterial pathogens via RNA-specific binding^45^,^46^,^47^,^48^,^49^,^50^. In pepper, the *RNA-binding protein 1* gene (*CaRBP1*) was identified as essential for HR and defense signaling in the cytoplasm^51^. However, plant RBPs that participate in the regulation of the defense response against fungal pathogen infection, especially in monocots, have not been reported prior to this work. This study is the first to identify wheat RBPs involved in host immunity during pathogen infection. Subsequent research is required to determine the functions of these RBPs.

### Chaperonins

Chaperones are the most prominent class of proteins that promote substrate protein folding; these proteins are usually classified according to their molecular weight^52^. Group I chaperonins are important components of chaperones known as GroEL-GroES in *Escherichia coli* and the heat shock proteins (HSPs) HSP60-HSP10 in eukaryotes^53^. In mammals, HSPs link the innate and adaptive immune systems; in circulation, these proteins serve as intercellular signals to the host^54^. In particular, HSP60 and HSP10 appear to be related to pregnancy, cancer, and autoimmune inhibition in association with each other^55^,^56^. *Mycobacterium tuberculosis* HSPl0 increases the rate of apoptosis in the mouse P19 teratocarcinoma cell line^57^. Evidence also suggested that human HSPl0 might be considered a pathogen-associated molecular pattern and damage-associated molecular pattern molecule to trigger Toll-like receptor signaling^55^. Combined with the results of the present study (Figs 7 and 8), plant HSP10 might play an important role in the positive regulation of the immune response as signaling molecules, whereas HSP60 might negatively regulate the defense reaction against pathogen infection. Further studies on the relationship of HSP10 and HSP60 for wheat defense response should be conducted in the future.

In addition, more than 80% spectra could not match the unique peptides or proteins (Table 3), whereas more than 200 regulated proteins were described as predicted proteins (Table S1), even when three database releases were used for protein identification^22^,^23^,^24^. This result was attributed to the huge and complex 17 Gbp hexaploid genome of wheat^24^, which has delayed wheat functional genomic research. This limitation also explains why research on wheat proteomics is rare.

## Methods

### Plant and pathogen

Wheat cultivar Su11 and *Pst* race CYR23 were used for incompatible reaction in this study. Su11 displays a typical HR upon infection with CYR23. The *Pst* isolate was maintained and propagated on a susceptible Chinese wheat cultivar, Mingxian 169. For biological stress treatments, the plants were grown, inoculated and maintained as previously described^58^. The *Pst*-inoculated leaves were sampled at 12, 24 and 48 hpi, and stored at −80 °C. Control plants were treated with sterile water.

### Protein preparation

For protein quantification, approximately 0.5 g fresh leaf tissue per sample was used to extract leaf total protein using the trichloroacetic acid-acetone precipitation method^59^. The cells were suspended in the Lysis buffer (7 mol/L Urea, 2 mol/L Thiourea, 4% CHAPS, 40 mmol/L Tris-HCl, pH 8.5, 1 mM PMSF, 2 mmol/L EDTA) and sonicated in ice. The proteins were reduced with 10 mmol/L DTT (final concentration) at 56 °C for 1 h and then alkylated by 55 mmol/L IAM (final concentration) in the darkroom for 1 h. The reduced and alkylated protein mixtures were precipitated by adding 4 × volume of chilled acetone at −20 °C for 2 hours. After centrifugation at 4 °C, 30,000 g, the pellet was dissolved in 0.5 mol/L TEAB (Applied Biosystems, Milan, Italy) and sonicated in ice. After centrifuging at 30,000 g at 4 °C, the supernatant was transferred to a new tube and quantified by using Bradford’s reagent (Sigma–Aldrich, St. Louis, MO, USA)^60^, and then kept at −80 °C for further analysis.

### iTRAQ Labeling and SCX fractionation

iTRAQ analysis was implemented at Beijing Genomics Institute (BGI, Shenzhen, China). Total protein (100 μg) was taken out of each sample solution and then the protein was digested with Trypsin Gold (Promega, Madison, WI, USA) with the ratio of protein: trypsin = 30: 1 at 37 °C for 16 h. After trypsin digestion, peptides were dried by vacuum centrifugation, and then reconstituted in 0.5 mol/L tetraethyl-ammonium bromide and processed according to the manufacture’s protocol for 8-plex iTRAQ reagent (Applied Biosystems). Briefly, one unit of iTRAQ reagent was thawed and reconstituted in 24 μL isopropanol. Samples were labeled with the iTRAQ tags as follow: Control-1 (113 tag), Su11-CYR23-12 hpi-1 (114 tag), Su11-CYR23-24 hpi-1 (115 tag), Su11-CYR23-48 hpi-1 (116 tag), Control-2 (117 tag), Su11-CYR23-12 hpi-2 (118 tag), Su11-CYR23-24 hpi-2 (119 tag), Su11-CYR23-48 hpi-2 (121 tag), respectively. The peptides were labeled with the isobaric tags, incubated at room temperature for 2 h. The labeled peptide mixtures were then pooled and dried by vacuum centrifugation.

SCX chromatography was performed with a LC-20AB HPLC Pump system (Shimadzu, Kyoto, Japan). The iTRAQ-labeled peptide mixtures were reconstituted with 4 mL buffer A (25 mmol/L NaH_2_PO_4_ in 25% ACN, pH 2.7) and loaded onto a 4.6 × 250 mm Ultremex SCX column containing 5-μm particles (Phenomenex). The peptides were eluted at a flow rate of 1 mL/min with a gradient of buffer A for 10 min, 5–60% buffer B (25 mmol/L NaH_2_PO_4_, 1 mol/L KCl in 25% ACN, pH 2.7) for 27 min, 60–100% buffer B for 1 min. The eluted peptides were pooled into 20 fractions, desalted with a Strata X C18 column (Phenomenex) and vacuum-dried.

### LC-ESI-MS/MS analysis

Each fraction was resuspended in buffer A (5% ACN, 0.1%FA) and centrifuged at 20,000 g for 10 min, the final concentration of peptide was about 0.5 μg/μL on average. 10 μL supernatant was loaded on a LC-20AD nanoHPLC (Shimadzu, Kyoto, Japan) by the autosampler onto a 2 cm C18 trap column. After that, the peptides were eluted onto a 10 cm analytical C18 column (inner diameter 75 μm) packed in-house. The samples were loaded at 8 μL/min for 4 min, then the 35 min gradient was run at 300 nL/min starting from 2 to 35% B (95% ACN, 0.1% FA), followed by 5 min linear gradient to 60%, then, followed by 2 min linear gradient to 80%, and maintenance at 80% B for 4 min, and finally return to 5% in 1 min.

The peptides were subjected to nanoelectrospray ionization followed by tandem mass spectrometry (MS/MS) in an Q EXACTIVE (Thermo Fisher Scientific, San Jose, CA) coupled online to the HPLC. Intact peptides were detected in the Orbitrap at a resolution of 70,000. Peptides were selected for MS/MS using high-energy collision dissociation (HCD) operating mode with a normalized collision energy setting of 27.0; ion fragments were detected in the Orbitrap at a resolution of 17,500. A data-dependent procedure that alternated between one MS scan followed by 15 MS/MS scans was applied for the 15 most abundant precursor ions above a threshold ion count of 20,000 in the MS survey scan with a following Dynamic Exclusion duration of 15 s. The electrospray voltage applied was 1.6 kV. For MS scans, the m/z scan range was 350 to 2,000 Da. For MS2 scans, the m/z scan range was 100–1,800. To account for biological variation and ensure only reproducible responses to treatments were selected, two independent biological replicate experiments were performed.

### Proteomic data analysis

Raw data files acquired from the Orbitrap were converted into MGF files using Proteome Discoverer 1.2 (PD 1.2, Thermo). Proteins identification was performed by using Mascot search engine (Matrix Science, London, UK; version 2.3.02). For protein identification, a mass tolerance of 20 ppm was permitted for intact peptide masses and 0.05 Da for fragmented ions, with allowance for one missed cleavages in the trypsin digests. The charge states of peptides were set to +2 and +3. To reduce the probability of false peptide identification, only peptides with significance scores ( ≥20) at the 99% confidence interval by a Mascot probability analysis greater than “identity” were counted as identified. And each confident protein identification involves at least one unique peptide.

For protein quantitation, it was required that a protein contains at least two unique peptides. The quantitative protein ratios were weighted and normalized by the median ratio in Mascot. We only used ratios with *p*-values < 0.05, and only fold changes of > 1.5 were considered as significant.

### Bioinformatics analysis

Functional annotations of the proteins were conducted using Blast2GO program against the non-redundant protein database (NR; NCBI) and three public wheat genome database, independently (http://gigadb.org/search/index/keyword/Triticum+aestivum/%20yt0/Search/file_page/22#result_files; http://www.ncbi.nlm.nih.gov/bioproject/PRJNA182347; http://wheat-urgi.versailles.inra.fr/Seq-Repository/). The KEGG database (http://www.genome.jp/kegg/) and the COG database (http://www.ncbi.nlm.nih.gov/COG/) were used to classify and group these identified proteins.

### RNA extraction, cDNA synthesis and qRT-PCR assay

Total RNA was extracted using Trizol Reagent (Life Technologies, Grand Island, NY, USA) according to the manufacturer’s instruction. Genomic DNA contaminants were removed by DNase I treatment. First-strand cDNA was synthesized using the M-MLV reverse transcriptase (Promega, Shenzhen, China) with an oligo-(dT_18_) primer. qRT-PCR was performed using a CFX96 real-time PCR detection system (Bio-Rad, Hercules, CA, USA). The primers for qRT-PCR are listed in Table S3. Relative gene quantification was performed as described in detail previously^58^ and normalized using the corresponding expression of the wheat elongation factor gene *TaEF-1a* (Genbank accession No. Q03033). All reactions were performed in triplicate, including three controls without the template.

### BSMV-mediated gene silencing

Seven special gene fragments were used to silence the transcription of selected proteins (Table S3). Linearized plasmids containing the tripartite BSMV genome were transcribed to RNA. Seven BSMV viruses were inoculated individually on the second leaf of the wheat seedlings at the two-leaf stage, as described previously^58^. After inoculation for 24 h in the dark, all treated seedlings were placed in a growth chamber at 25 ± 2 °C and then examined for symptoms. BSMV:TaPDS was used as a negative control^61^. Control plants were treated with full-strength inoculation buffer. The fourth leaf of each plant was then inoculated with fresh urediniospores of CYR23 or CYR32 at 12 days after the viral inoculation, and these leaves sampled at 0, 12, 24 and 48 hpi for RNA isolation to evaluates the silencing efficiencies of selected genes using qRT-PCR. The infection types of stripe rust were examined at 14 dpi. The experiment was repeated three times.

### Statistical analysis

Analysis of variance (ANOVA) was performed to determine the significant differences between each treatment using SAS (version 8.12; SAS Institute Inc., Cary, NC, USA). Duncan’s multiple range tests were used for multiple comparison tests.

## Additional Information

**How to cite this article**: Yang, Y. *et al.* Quantitative Proteomics Reveals the Defense Response of Wheat against *Puccinia striiformis* f. sp. *tritici. Sci. Rep.* **6**, 34261; doi: 10.1038/srep34261 (2016).

## Supplementary Material

Supplementary Information

Supplementary Table S1

Supplementary Table S2

## Figures and Tables

**Table 1:** Summary of identified proteins regulated by avirulent *Puccinia striiformis* f. sp. *tritici*.

Group	Total spectra	Spectra	Unique spectra	Peptide	Unique peptide	Protein
*Aegilops tauschii*	288,769	55,806	50,519	4,729	4,373	1,774
*Triticum urartu*		15,502	11,325	953	915	338
*Triticum aestivum*		52,473	45,248	3,996	3,683	1,363

**Table 2:** The numbers of differentially regulated wheat proteins in three treatment groups (12, 24, and 48 hpi)by avirulent *Puccinia striiformis* f. sp. *tritici*.

Group	Up-regulated			Down-regulated		
	12 hpi	24 hpi	48 hpi	12 hpi	24 hpi	48 hpi
*Aegilops tauschii*	7	171	38	28	58	29
*Triticum urartu*	1	29	8	7	17	6
*Triticum aestivum*	7	122	30	19	35	22

hpi, hour post-inoculation.

**Table 3:** Protein groups with remarkable differential accumulation in response to avirulent *Puccinia striiformis* f. sp. *tritici* infection.

Accession No.	Database	Description
*ROS metabolism*		
AEGTA00133	*Aegilops tauschii*	cytosolic Cu/Zn superoxide dismutase
AEGTA05616	*Ae. tauschii*	glutathione peroxidase 1
AEGTA06591	*Ae. tauschii*	peroxidase
AEGTA07275	*Ae. tauschii*	NADPH2:quinone reductase
AEGTA07473	*Ae. tauschii*	peroxidase
AEGTA08745	*Ae. tauschii*	class III peroxidase
AEGTA09652	*Ae. tauschii*	peroxidase
AEGTA10714	*Ae. tauschii*	peroxidase
AEGTA18921	*Ae. tauschii*	peroxidase
AEGTA24337	*Ae. tauschii*	class III peroxidase
AEGTA26193	*Ae. tauschii*	glutathione peroxidase
AEGTA26338	*Ae. tauschii*	phospholipid hydroperoxide glutathione peroxidase-like protein
AEGTA28018	*Ae. tauschii*	peroxidase
AEGTA29816	*Ae. tauschii*	L-ascorbate peroxidase
AEGTA29877	*Ae. tauschii*	Cu/Zn superoxide dismutase
AEGTA30142	*Ae. tauschii*	peroxidase 4
AEGTA30520	*Ae. tauschii*	peroxidase
AEGTA31232	*Ae. tauschii*	thioredoxin-dependent peroxidase
AEGTA31382	*Ae. tauschii*	NADPH2:quinone reductase
AEGTA32143	*Ae. tauschii*	PREDICTED: 2-Cys peroxiredoxin BAS1, chloroplastic-like
AEGTA43716	*Ae. tauschii*	peroxidase 8
Contig85551	*Ae. tauschii*	plastid thylakoid-bound ascorbate peroxidase, partial
TRAES3BF072500100CFD_c1	*Triticum aestivum*	peroxidase 8
TRAES3BF102700090CFD_c1	*T. aestivum*	PREDICTED: peroxiredoxin-2F, mitochondrial-like
gi|473781575|gb|EMS46089.1|	*T. urartu*	L-ascorbate peroxidase
gi|473817674|gb|EMS46926.1|	*T. urartu*	plastid thylakoid-bound ascorbate peroxidase, partial
gi|473835923|gb|EMS47195.1|	*T. urartu*	peroxidase
gi|473949385|gb|EMS50650.1|	*T. urartu*	peroxidase 6
gi|473958051|gb|EMS51034.1|	*T. urartu*	catalase
gi|473960762|gb|EMS51084.1|	*T. urartu*	peroxidase 5
gi|474012122|gb|EMS52565.1|	*T. urartu*	PREDICTED: peroxidase 54-like [Brachypodium distachyon]
gi|474057823|gb|EMS53898.1|	*T. urartu*	glutathione peroxidase, partial
gi|474066770|gb|EMS54178.1|	*T. urartu*	quinone reductase
gi|474074307|gb|EMS54484.1|	*T. urartu*	peroxidase 8
gi|474139638|gb|EMS56485.1|	*T. urartu*	PREDICTED: peroxiredoxin-2F, mitochondrial-like
gi|474145957|gb|EMS56757.1|	*T. urartu*	thioredoxin-dependent peroxidase
gi|474159921|gb|EMS57248.1|	*T. urartu*	peroxidase 3
gi|474183470|gb|EMS57821.1|	*T. urartu*	predicted protein
gi|474249697|gb|EMS60130.1|	*T. urartu*	PREDICTED: peroxidase 5-like
gi|474256124|gb|EMS60363.1|	*T. urartu*	Superoxide dismutase 2
gi|474292610|gb|EMS61345.1|	*T. urartu*	putative Td650 protein
gi|474413223|gb|EMS66985.1|	*T. urartu*	predicted protein
*Peptidyl-prolyl cis-trans isomerases*		
AEGTA02200	*Ae. tauschii*	FKBP-type peptidyl-prolyl *cis-trans* isomerases 1 PPIases
AEGTA05000	*Ae. tauschii*	FKBP-type peptidyl-prolyl *cis-trans* isomerases 1 PPIases
AEGTA06390	*Ae. tauschii*	Peptidyl-prolyl *cis-trans* isomerase (rotamase) - cyclophilin family
AEGTA08970	*Ae. tauschii*	FKBP-type peptidyl-prolyl *cis-trans* isomerases 1 PPIases
AEGTA26095	*Ae. tauschii*	Peptidyl-prolyl *cis-trans* isomerase (rotamase) - cyclophilin family
AEGTA27753	*Ae. tauschii*	FKBP-type peptidyl-prolyl *cis-trans* isomerases 1 PPIases
AEGTA31129	*Ae. tauschii*	Peptidyl-prolyl *cis-trans* isomerase (rotamase) - cyclophilin family
AEGTA32057	*Ae. tauschii*	FKBP-type peptidyl-prolyl *cis-trans* isomerases 1
gi|473907428|gb|EMS49431.1|	*T. urartu*	FKBP-type peptidyl-prolyl *cis-trans* isomerases 1
gi|473926457|gb|EMS49826.1|	*T. urartu*	Peptidyl-prolyl *cis-trans* isomerase (rotamase) - cyclophilin family
gi|473961253|gb|EMS51102.1|	*T. urartu*	Peptidyl-prolyl *cis-trans* isomerase (rotamase) - cyclophilin family
gi|474142167|gb|EMS56572.1|	*T. urartu*	FKBP-type peptidyl-prolyl *cis-trans* isomerases 1
*RNA-binding proteins*		
AEGTA10070	*Ae. tauschii*	RNA-binding proteins (RRM domain)
AEGTA10595	*Ae. tauschii*	RNA-binding proteins (RRM domain)
AEGTA28197	*Ae. tauschii*	RNA-binding proteins (RRM domain)
AEGTA28246	*Ae. tauschii*	RNA-binding proteins (RRM domain)
AEGTA28251	*Ae. tauschii*	RNA-binding proteins (RRM domain)
AEGTA28395	*Ae. tauschii*	RNA-binding proteins (RRM domain)
AEGTA30690	*Ae. tauschii*	RNA-binding proteins (RRM domain)
TRAES3BF046400050CFD_c1	*T. aestivum*	RNA-binding proteins (RRM domain)
TRAES3BF080700020CFD_c1	*T. aestivum*	RNA-binding proteins (RRM domain)
TRAES3BF152900030CFD_c1	*T. aestivum*	RNA-binding proteins (RRM domain)
gi|473997750|gb|EMS52114.1|	*T. urartu*	RNA-binding proteins (RRM domain)
gi|474191086|gb|EMS58171.1|	*T. urartu*	RNA-binding proteins (RRM domain)
gi|474264748|gb|EMS60656.1|	*T. urartu*	RNA-binding proteins (RRM domain)
*Chaperonins*		
AEGTA27057	*Ae. tauschii*	Co-chaperonin GroES (HSP10)
AEGTA28112	*Ae. tauschii*	Co-chaperonin GroES (HSP10)
AEGTA28933	*Ae. tauschii*	Co-chaperonin GroES (HSP10)
AEGTA32594	*Ae. tauschii*	Co-chaperonin GroES (HSP10)
AEGTA06357	*Ae. tauschii*	Chaperonin GroEL (HSP60 family)
gi|474209261|gb|EMS58795.1|	*T. urartu*	Co-chaperonin GroES (HSP10)
gi|474407512|gb|EMS66632.1|	*T. urartu*	Co-chaperonin GroES (HSP10)
