# Corrigendum: Quantitative Proteomics Reveals the Defense Response of Wheat against *Puccinia striiformis* f. sp. *tritici*

**DOI:** 10.1038/srep38464

**Published:** 2016-12-22

**Authors:** Yuheng Yang, Yang Yu, Chaowei Bi, Zhensheng Kang

Scientific Reports 6: Article number: 3426110.1038/srep34261; published online: 09 28 2016; updated: 12 22 2016

This Article contains an error in the Acknowledgements section.

“This work was supported by the Fundamental Research Funds for the Central Universities (XDJK2016A020, XDJK2015C060, SWU114046, 2362015xk04), the Fundamental and Advanced Research Projects of Chongqing City (cstc2016jcyjA0316), the Open Project Program of State Key Laboratory of Crop Stress Biology for Arid Areas (CSBAA2015009), and the Visiting Scholar Funds of Key Laboratory of Plant Protection Resources and Pest Management of Ministry of Education. We thank anonymous reviewers for their kind suggestions.”

should read:

“This work was supported by the Fundamental Research Funds for the Central Universities (XDJK2016A020, XDJK2015C057, SWU114046, 2362015xk04), the Fundamental and Advanced Research Projects of Chongqing City (cstc2016jcyjA0316), the Chongqing Postdoctoral Science Foundation (Xm2016124), the Open Project Program of State Key Laboratory of Crop Stress Biology for Arid Areas (CSBAA2015009), and the Visiting Scholar Funds of Key Laboratory of Plant Protection Resources and Pest Management of Ministry of Education. We thank anonymous reviewers for their kind suggestions.”

