# Correction: Quantitative Proteomics Reveals the Defense Response of Wheat against *Puccinia striiformis* f. sp. *tritici*

**DOI:** 10.1038/s41598-026-66320-8

**Published:** 2026-08-21

**Authors:** Yuheng Yang, Yang Yu, Chaowei Bi, Zhensheng Kang

**Affiliations:** 1 College of Plant Protection, Southwest University, Beibei, Chongqing, 400715 P.R. China; 2 Key Laboratory of Plant Protection Resources and Pest Management of Ministry of Education, State Key Laboratory of Crop Stress Biology for Arid Areas and College of Plant Protection, Northwest A&F University, Yangling, Shaanxi 712100 P.R. China

Correction to: *Scientific Reports* 10.1038/srep34261, published online 28 September 2016

This Article contains errors in panel B of Fig. 10.

As a result of an oversight during figure assembly, the images “**BSMV: AEGTA28112” for “CYR32”** and “**BSMV: AEGTA32594” for “CYR32”** are duplicated.

The correct Fig. 10 and accompanying legend appear below.

**Fig. 10:**
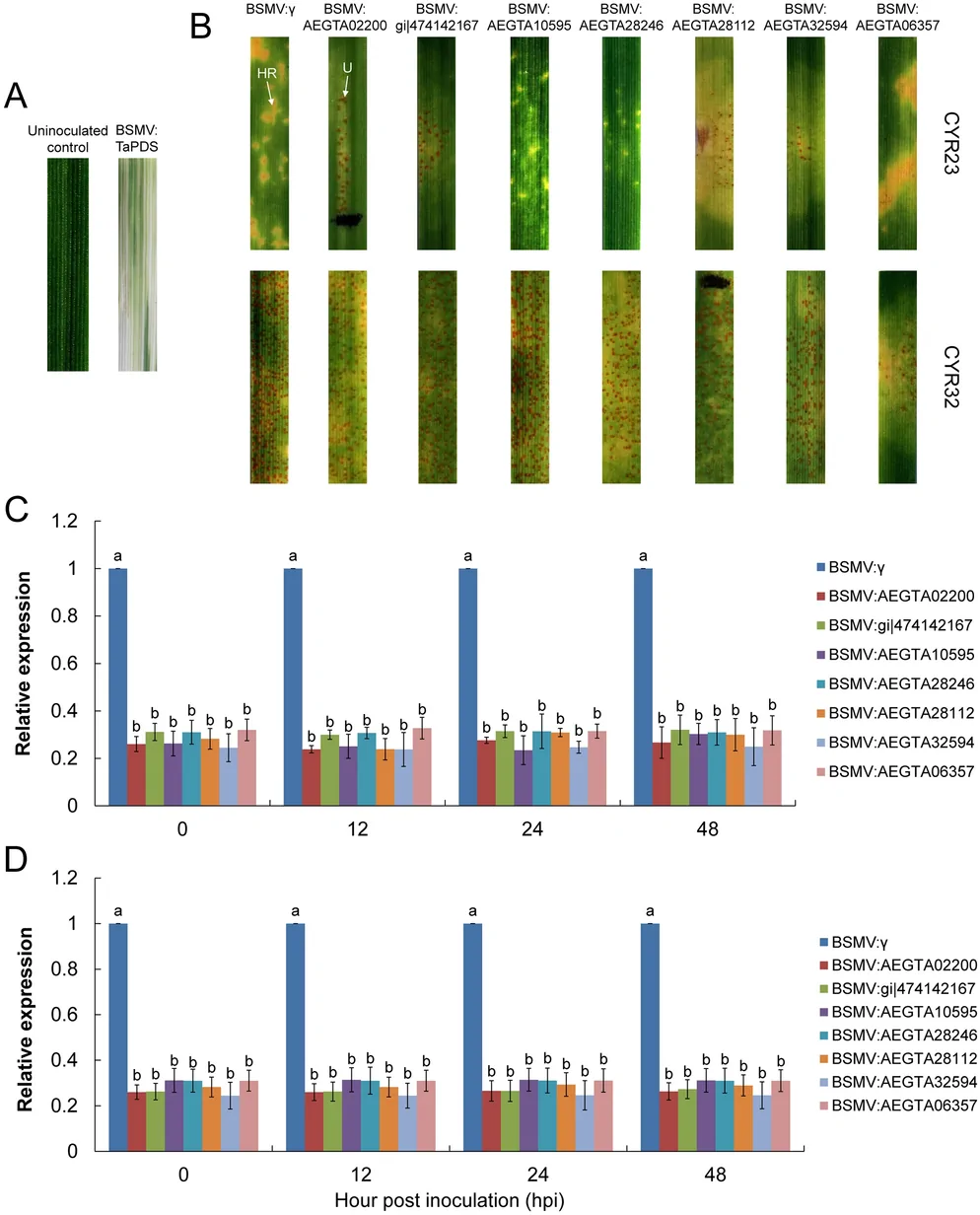
Functional characterization of differentially expressed proteins by BSMV-VIGS system. (**A**) Mild chlorotic mosaic symptoms were observed on the fourth leaves inoculated with BSMV:TaPDS at 12 dpi; uninoculated control, wheat leaves treated with full-strength inoculation buffer. (**B**) Disease symptoms were observed at 14 dpi on the fourth leaves of wheat plants that were inoculated with the avirulent pathogen CYR23 and virulent pathogen CYR32, respectively. HR, hypersensitive response; U, uredium. (**C**) Relative transcript levels of differentially expressed proteins assayed in knocked-down wheat leaves inoculation with CYR23. (**D**) Relative transcript levels of differentially expressed proteins assayed in knocked-down wheat leaves inoculation with CYR32. The mean value and standard deviation of gene expression were calculated from three independent biological replications. ANOVA was conducted to determine the differences between each time point. Superscripts with the same letter indicate that values are not significantly different at *p* < 0.01.

