# Supplementary material for: Quantitative Proteomics Reveals the Defense Response of Wheat against Puccinia striiformis f. sp. tritici
Source: Sci Rep. 2016 Sep 28;6:34261. doi: 10.1038/srep34261 (PMC5039691; doi:10.1038/srep34261)
Supplement: Supplementary Information [file srep34261-s1.pdf]

Quantitative Proteomics Reveals the Defense Response of Wheat against *Puccinia striiformis* f. sp. *tritici*

Yuheng Yang[1,2,*], *Yang Yu*[1], *Chaowei Bi*[1], *Zhensheng Kang*[2,*]

Supplementary information

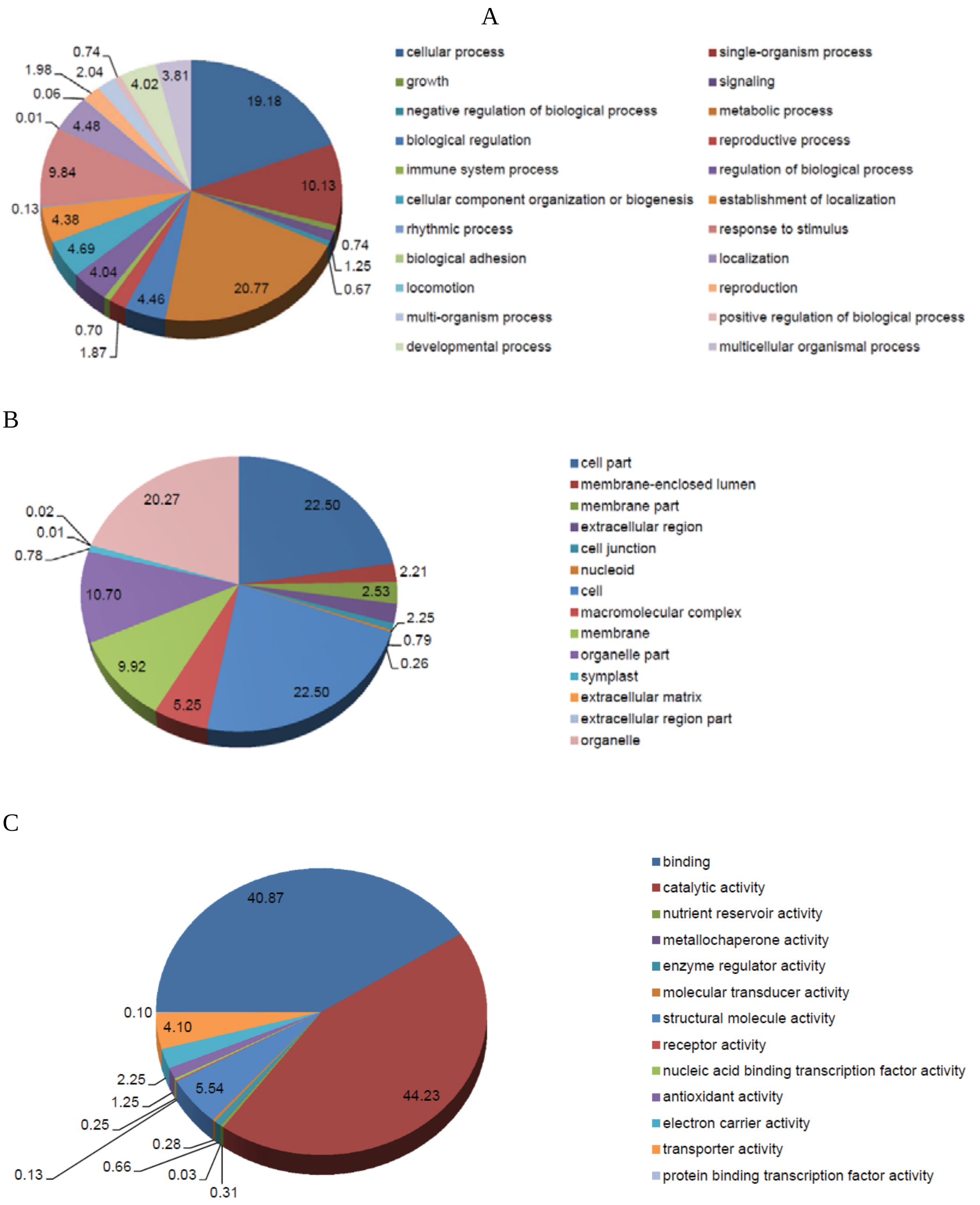

Figure S1. Gene ontology analysis of the identified proteins in the wheat–*Puccinia striiformis* f. sp. *tritici* incompatible interaction. The identified proteins were classified into the (**A**) biological process, (**B**) cellular component, and (**C**) molecular function categories.

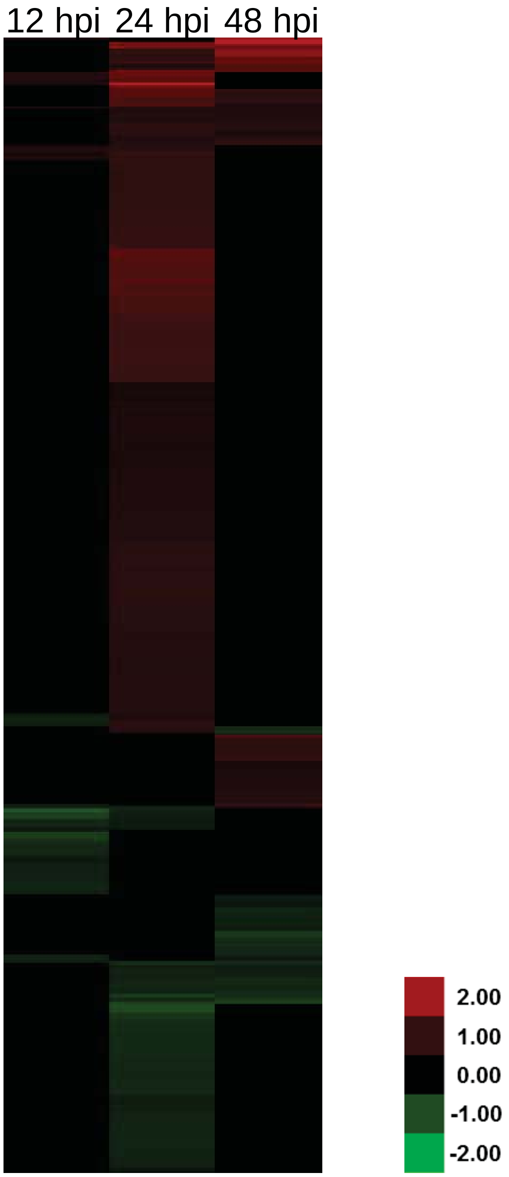

Figure S2. Heat map representation of 530 differentially accumulated wheat proteins induced by *Puccinia striiformis* f. sp. *tritici*. Red color indicated high expression whereas green color indicated low expression.

Table S3. Primers used in this study.

**Primer name**	**Primer sequence (5'-3')**		**Description**
AEGTA02200	Forward	TTCACTACCGTGGAACACTTAC	FKBP-type peptidyl-prolyl *cis-trans* isomerases 1
	Reverse	ATTCCCTGATCCCATCCTTTG	
gi|474142167|gb|EMS56572.1|	Forward	GAGGCCAGAGGTTGGTTATT	FKBP-type peptidyl-prolyl *cis-trans* isomerases 1
	Reverse	AGTTCGACATCCAGCTCAATAG	
AEGTA10595	Forward	TTGGTGCCAGGGTAATCTTC	RNA-binding protein (RRM domain)
	Reverse	GCAGCCTTTGCATCTTCTATTG	
AEGTA28246	Forward	ACTCCAAAGTCCAAGGGATATG	RNA-binding protein (RRM domain)
	Reverse	TATGCTCTATTCACACGGATGG	
AEGTA28112	Forward	AGGCAATTCCGTTCTGTACTC	Co-chaperonin GroES (HSP10)
	Reverse	AGGATAGCCATCAGGTCAGATA	
AEGTA32594	Forward	CGCTGGTACTGAGGTTGAATAC	Co-chaperonin GroES (HSP10)
	Reverse	CCTTGATGAGAATCCGGTCATT	
AEGTA06357	Forward	TGAGCTACCCTACCACACTAATA	Chaperonin GroEL (HSP60 family)
	Reverse	GTTGGCAGCATTGTATCCATAAC	
BSMV:AEGTA02200	Forward	ctaGCTAGCTCCACTGCTGAAGATAACTG	Construction of BSMV:AEGTA02200 for
	Reverse	ctaGCTAGCTCCTGACTTGTAGACACTTG	silencing AEGTA02200
BSMV:gi|474142167	Forward	ctaGCTAGCGTGTCGCAGTACATTAC	Construction of BSMV:gi|474142167 for
	Reverse	ctaGCTAGCTCAGGAGGAACAATAACCAA	silencing gi|474142167
BSMV:AEGTA10595	Forward	ctaGCTAGCTCTCACTTCAACCTCAACAA	Construction of BSMV:AEGTA10595 for
	Reverse	ctaGCTAGCTTCCTCCACGTACTCCT	silencing AEGTA10595
BSMV:AEGTA28246	Forward	ctaGCTAGCGCCATTGAGAGCCTGAAT	Construction of BSMV:AEGTA28246 for
	Reverse	ctaGCTAGCTTGAGCACTTCCGTTGT	silencing AEGTA28246
BSMV:AEGTA28112	Forward	ctaGCTAGCATGGAGGTGGAGTTGAATG	Construction of BSMV:AEGTA28112 for
	Reverse	ctaGCTAGCAAGAACACGGTCGCTAAG	silencing AEGTA28112
BSMV:AEGTA32594	Forward	ctaGCTAGCTGAGATTGTTGCTGTTGGA	Construction of BSMV:AEGTA32594 for
	Reverse	ctaGCTAGCAGATGCTTGGAGTTGTTGT	silencing AEGTA32594
BSMV:AEGTA06357	Forward	ctaGCTAGCGTGCTCGCCAACAAGTAT	Construction of BSMV:AEGTA06357 for
	Reverse	ctaGCTAGCAAGAACCTTCATTCCTTCAG	silencing AEGTA06357
TaEF-1α	Forward	TGGTGTCATCAAGCCTGGTATGGT	wheat elongation factor 1α
	Reverse	ACTCATGGTGCATCTCAACGGACT	
